# Effect of statins on risk and mortality of urologic malignancies: Protocol of an umbrella review

**DOI:** 10.1371/journal.pone.0264076

**Published:** 2022-03-02

**Authors:** Xinyu Zhai, Pengsheng Yi, Xitao Wang, Haifeng Wang, Xuejun Yang, Zubing Mei, Minyao Ge

**Affiliations:** 1 Department of Urology, Shuguang Hospital, Shanghai University of Traditional Chinese Medicine, Shanghai, China; 2 Department of Hepato-Biliary-Pancrease, Affiliated Hospital of North Sichuan Medical College, Nanchong, Sichuan Province, China; 3 Department of Surgery, Binhai New Area Hospital of Traditional Chinese Medicine, Tianjin, China; 4 Department of Urology, Shanghai East Hospital, Tongji University School of Medicine, Shanghai, China; 5 Institute of Nephrology, Shuguang Hospital Affiliated to Shanghai University of Traditional Chinese Medicine, Shanghai, China; 6 Anorectal Disease Institute of Shuguang Hospital, Shanghai, China; 7 Department of Anorectal Surgery, Shuguang Hospital, Shanghai University of Traditional Chinese Medicine, Shanghai, China; Universita degli Studi di Brescia, ITALY

## Abstract

**Introduction:**

Urologic malignancies are the major causes of morbidity and mortality in men over 40 years old, accounting for more than 20% of all malignant tumors. Several meta-analyses are shown that statin exposure can reduce the morbidity and mortality of various urologic cancers. The adjuvant roles of statin in tumor prevention and anti-tumor activity are now being gradually recognized and have gained attention. Nevertheless, to date, multiple clinical studies and meta-analyses found inconsistent results of their anti-cancer effects. This study aims to evaluate the credibility of the published systematic reviews and meta-analyses that assessed the effects of statin exposure for the incidence and mortality of urologic cancers through an umbrella review.

**Methods and analysis:**

The guidance of overviews of systematic reviews reported in the Cochrane Handbook for Systematic Reviews of interventions will be followed while performing and reporting this umbrella review. This project was registered in PROSPERO with the registration number of CRD42020208854. PubMed, Embase and Cochrane Library will be searched for systematic reviews to identify and appraise systematic reviews or meta-analyses of interventional and observational studies examining statin use and the risks of urologic cancer incidence and mortality without language restriction. The search will be carried out on 10 February 2022. Systematic reviews based on qualitative, quantitative or mixed-methods studies will be involved and critically evaluated by two authors using the Assessment of Multiple Systematic Reviews 2 (AMSTAR2, an updated version of AMSTAR) tool. We will determine the level of evidence using the GRADE (Grading of Recommendations, Assessment, Development, and Evaluations) tool. The summary effect estimates will be calculated using random-effects models. Between- study heterogeneity will be assessed using the I^2^ statistic. Furthermore, we will also assess the evidence of excess significance bias and evidence of small study effects.

**Ethics and dissemination:**

Ethics approval is not required as we will search and gather data based on the published systematic reviews and meta-analyses. We plan to publish the results of this umbrella review in a peer-reviewed journal and will be presented at a urological disease conference. All the relevant additional data will also be uploaded to the online open access databases.

**PROSPERO registration number:**

CRD42020208854.

## Introduction

Malignant tumors of the urinary system, named urologic malignancies mainly include prostate cancer, urothelial cancer, kidney cancer, testicular cancer and other urinary organ malignancies, which are the major causes of morbidity and mortality in men over 40 years old, accounting for more than 20% of all malignant tumors [1]. Prostate cancer is the second most common male malignant tumor in the world. There is no doubt that urologic cancers are a major burden on health care systems and individual patients worldwide [2]. Although current immunotherapy strategies such as therapeutic vaccines or immune checkpoint inhibitors have greatly improved the prognosis of these patients [3], the global mortality rate of urinary cancer is still extremely high [4–6]. It was estimated that 375,304 and 212,536 people died of prostate and bladder cancer in 2020, respectively [7].

Statins have been widely used for reducing cardiovascular events in both the primary and secondary prevention of cardiovascular disease. It is estimated that from 2008 to 2018, global use of lipid-modifying agents (LMAs), especially statins, was growing at an annual rate of about 4.13%. In 2018, more than 3% populations (173 million people) were estimated to take daily LMA all around the world [8]. Moreover, recent guidelines have defined the scope of use of statin more widely [9]. Because of the worldwide increase in statin use in the past few years [10–12], the potential effects of these types of medications on the antineoplastic properties have attracted great attention [13, 14]. The role of statins in the prevention and adjuvant therapeutic effect on various tumors are indicated by some clinical evidence [15–18]. Many recent meta-analyses have claimed that statins also are associated with outcomes of several neoplasms, such as ovarian, breast, prostate and colorectal cancer [19–23]. However, these studies and meta-analyses varied in the strength of evidence which added limited value to guide clinical practice. Therefore, in this study we aim to perform an umbrella review to summarize the literature and to evaluate the validity and credibility of the evidence regarding the effect of statin intake on risk and mortality of urologic malignancies.

## Methods and analysis

### Protocol registration

We will strictly follow the Preferred Reporting Items for Systematic Review and Meta-Analysis Protocols (PRISMA-P) guidelines in the conduct of this umbrella review based on the predesigned protocol [24] (**S1 Table**). Details of the umbrella review can be obtained on PROSPERO (www.crd.york.ac.uk/prospero), which has been registered and assigned the registration number of CRD42020208854. The review is anticipated to be carried out between February, 2022 and June 2022.

### Data sources and search strategies

As was reported by previously reports [24–26], the methodology of umbrella review will be followed to critically evaluate all available systematic reviews on the topic. Umbrella review is a kind of review which includes systematic reviews or evidence syntheses as study type used to examine the level of evidence for clinical practice [27]. Three commonly used databases including PubMed, Embase and the Cochrane Database of Systematic Reviews will be carried out on 10 February 2022. Eligible published systematic reviews or meta-analyses of interventional and observational studies on the associations between statin use and risk of urologic malignancies or mortality after the diagnosis of urologic malignancies will be involved (detailed search strategy for Pubmed in **Table 1**). Systematic reviews or meta-analyses of clinical interventional studies investigating outcomes of statin therapy or the added effect of statins on a certain therapy will also be included. Of note, we will not restrict the search strategy to meta-analyses published in English.

**Table 1:** Search strategy for pubmed database.

**Search strategy**
***Statin terms*:**
1 "Hydroxymethylglutaryl-CoA Reductase Inhibitors"[Mesh]
2 "Simvastatin"[Mesh]
3 (statin or statins or atorvastatin or cerivastatin or fluvastatin or lovastatin or pravastatin or
simvastatin or lipitor or baycol or lescol or mevacor or altocor or pravachol or lipostat or zocor or
mevinolin or compactin or fluindostatin or rosuvastatin) [Title/Abstract]
4 or/1-3
***Urologic cancer terms*:**
5 "Urologic Neoplasms"[Mesh]
6 "Kidney Neoplasms"[Mesh]
7 "Carcinoma, Renal Cell"[Mesh]
8 "Carcinoma, Transitional Cell"[Mesh]
9 "Ureteral Neoplasms"[Mesh]
10 "Urinary Bladder Neoplasms"[Mesh]
11 "Prostatic Neoplasms"[Mesh]
12 "Urogenital Neoplasms"[Mesh]
13 ((Urologic or Kidney or Renal Cell or Transitional Cell or Ureteral or Bladder or Prostat* or
Urogenital) and (cancer* or oncolog* or neoplasm* or carcinom* or tumor* or tumour* or
malignan*))[Title/Abstract]
14 or/5-13
***Umbrella review terms*:**
15 "Systematic Review" [Publication Type]
16 "Meta-Analysis" [Publication Type]
17 "Meta-Analysis as Topic"[Mesh]
18 "Systematic Reviews as Topic"[Mesh]
19 (systematic review or meta-analysis or metaanalysis)[Title/Abstract]
20 or/15-19
***Final search results*: *Combining Statin and Urologic cancer and Umbrella review*:**
18 4 and 14 and 20

### Study selection criteria

The followings will be the eligibility criteria for our umbrella review:

### Study design

Systematic reviews with meta-analyses of observational studies (including prospective or retrospective population-based, community-based or hospital-based cohort studies, case-control /nested case-control studies) and randomized controlled trials (RCTs) will be included.

### Intervention/exposure

Statin medications will be involved as intervention measure or exposure.

### Outcomes

All-cause mortality, cause-specific mortality, and risk of urologic malignancies including bladder, kidney, prostate, testicular, ureteral, urethral, penile, adrenal and upper urinary tract cancer will be assigned as the main study outcomes.

We will also include meta-analyses that summarized all RCTs, prospective and retrospective cohort/case-control studies in their analyses. If more than one meta-analysis published on the same topic with one of the same outcomes, we will select the one with the largest number of original studies for review. Systematic reviews with observational studies and RCTs will be analyzed separately. We will exclude systematic reviews without meta-analysis. Furthermore, meta-analyses involving studies with unadjusted effect estimates will also be excluded.

### Literature screening and data extraction

All of the retrieving citation records will be imported to EndNote X9 (Thomson Reuters, Toronto, Ontario, Canada) to remove duplicates. Two reviewers will be involved in the literature screening by title, abstract and full text reading. Any discrepancies will be resolved through discussion or by a supervising author until consensus is reached.

Two reviewers will extract data from each study and a third author will double check the extracted data. For each published meta-analysis, the following data will be extracted: name of the first author, publication year, type of exposure, dose and duration of statin exposure, number of included studies, study design of the original studies, sample size of total population and events, effect estimates and 95% confidence interval, follow-up period for cohort studies, quality score or risk of bias of original studies (mean/median) if reported, publication bias (p values of Egger’s or Begg’s test), funding information, and reported conflict of interest.

### Critical appraisal and quality of evidence evaluation

Two reviewers will assess the methodological quality of the included systematic reviews using the Assessment of Multiple Systematic Reviews 2 (AMSTAR2) updated version. This tool is a 16-item checklist developed to rate the methodological quality of each systematic review. For the quality of the original studies included in the meta-analyses, we will use the Newcastle Ottawa Scale (NOS) or the Cochrane risk of bias tool to assess their risk of bias. We will also use the GRADE (Grading of Recommendations, Assessment, Development and Evaluation) system to assess the quality of evidence for each outcome involved in the umbrella review, which divides the evidence into “high”, “moderate”, “low” and “very low” quality [28].

### Data synthesis and analysis

#### Estimation of summary effect and prediction intervals

All analyses will be carried out using Stata 12.0 software (StataCorp, TX, USA). For each association of statin use with risk or mortality of urological malignancies, we will recalculate the selected meta-analysis using risk estimates of the primary studies included in the published meta-analyses that adjusted for the most confounders.

We will recalculate the effect estimates and corresponding 95% confidence intervals (CIs) by using the DerSimonian and Laird random effects model, considering the within-study and between-study heterogeneity [29]. 95% prediction intervals (PIs) for the combined random effects estimates will also be calculated, which can further account for between-study heterogeneity and can also imply the range for the effects that will be expected in future studies investigating that same associations [30].

#### Assessment of between-study heterogeneity

Between-study heterogeneity will be assessed with Cochran’s Q test and I^2^ statistic, which can reflect either genuine between-study heterogeneity or bias. The < 25%, 25% ≤ I^2^ ≤ 50%, I^2^ > 50% are considered low, moderate and high heterogeneity, respectively [31].

#### Assessment of publication bias, small study effects and excess significance biases

Publication bias and small study effects will be assessed for each outcome of the meta-analysis through funnel plot symmetry and Egger’s regression test [32, 33]. Small study effects mean that smaller studies tend to generate larger risk estimates than larger studies. The Egger’s regression test with a P value less than 0.10 in random-effects meta-analysis will be taken as statistical evidence of the existence of small study effects or potential publication bias [32]. The excess significance test will be applied to evaluate whether the observed (O) number of studies with statistically significant results (P<0.05 for positive studies) is greater than the expected (E) number of positive studies by using a χ2 test. The threshold of excess significance bias is set at P<0.10 [34, 35].

#### Assessment of epidemiological credibility

We will only identify and report the most credible and unbiased associations or effects. The association between statin use and risk or mortality of urologic malignancies is judged as ‘convincing’ if the association yielded using random-effects model is significant with a P value less than 10^−6^, the meta-analysis has more than 1000 cases, has small between-study heterogeneity (I^2^< 50%), the 95% PI excludes the null value and there is no evidence of small-study effects or excess significance of bias [36].

We will assume as ‘highly suggestive’ the associations where the effect is significant with a P value less than 10^−6^ using random-effect model, has more than 1000 cases and the largest study shows a statistically significant effect with P value less than 0.05 [37]. We will judge as ‘suggestive’ the associations with significant effect at a P value less than 10^−3^ and more than 1000 cases. Other associations with a P value less than 0.05 and non-significant association at a P value less than 0.05 will be grouped as ‘weak’ associations.

## Discussion

This umbrella review of systematic reviews of interventional and observational studies will provide a comprehensive overview and generate a clear hierarchy of evidence on the statin use associated with risk or mortality of urologic malignancies.

To the best of our knowledge, this will be the first umbrella review addressing this topic. From a clinical point of view, it is valuable to have an in-depth understanding of the relationship between statin use and the risk of urinary tumors and whether it affects the death of patients with urinary tumors in that it can provide predictive information for people who use statins or patients with urologic tumors, as well as clinicians. Our umbrella review may also help to reveal the mechanism of statins in the prevention and treatment of urinary tumors and propose possible treatment approaches in the future (for example, guidance and advice on the use of statins in people with hyperlipidemia or patients with urinary tumors).

Our umbrella review has several strengths. First, PRISMA guidelines will be rigorously followed in the conduct of the review and the methodological quality of the eligible meta-analyses included in the umbrella review will be assessed with the AMSTAR 2 tool, which can guarantee the adequate methodological quality of systematic reviews and ensure reliable findings. Second, beyond summarizing the findings for risk and mortality outcomes, we will further explore the extent of bias and heterogeneity in the observational and interventional statin literature. Third, multiple outcome measures will be involved. As a matter of fact, we will try to explore the effects of statins on multiple outcome measures including both the risk and mortality of urologic malignancies in this umbrella review. Finally, our umbrella review will include systematic reviews published in several languages, if available, to avoid the potential of publication bias.

However, one major limitation of this study is that a small number of systematic review studies will be anticipated to be involved for analysis given the relative novelty of the related studies on this topic. Therefore, it will lead us to a future direction regarding this topic on more efforts to address the gaps associated with more high-quality evidence.

## Supporting information

S1 Table PRISMA-P checklist. (DOC)

## Abbreviations

AMSTAR2: Assessment of Multiple Systematic Reviews 2
CI: confidence interval
HR: hazard ratio
NOS: Newcastle Ottawa Scale
PI: prediction interval
PRISMA-P: Preferred Reporting Items for Systematic Review and Meta-Analysis Protocols
OR: odd ratio
RCT: randomized controlled trials
RR: relative risk
